# Comprehensive analysis of serum tumor markers and *BRCA1/2* germline mutations in Chinese ovarian cancer patients

**DOI:** 10.1002/mgg3.672

**Published:** 2019-04-10

**Authors:** Hongyu Deng, Ming Chen, Xinwu Guo, Jianfu Heng, Xunxun Xu, Limin Peng, Hui Jiang, Guoli Li, Julia X. Day, Jinliang Li, Dongyong Shan, Yinghua Li, Yanjie Zhou, Bin Liu, Lizhong Dai, Xiaochun Wang, Jun Wang

**Affiliations:** ^1^ Department of Laboratory Medicine, Xiangya School of Medicine Central South University Changsha China; ^2^ Hunan Cancer Hospital & The Affiliated Cancer Hospital of Xiangya School of Medicine Central South University Changsha China; ^3^ Sanway Gene Technology Inc. Changsha China; ^4^ School of Life Sciences Central South University Changsha China; ^5^ Department of Oncology The Third Xiangya Hospital of Central South University Changsha China; ^6^ Department of Obstetrics and Gynecology Dalian Municipal Central Hospital Affiliated to Dalian Medical University Dalian China

**Keywords:** *BRCA1/2 *mutation, next‐generation sequencing, ovarian cancer, serum tumor markers

## Abstract

**Background:**

The serum tumor markers has been widely used in ovarian cancer diagnosis. *BRCA1/2* germline mutations are the most common predisposing factors for ovarian cancer development. This study aimed to comprehensively investigate serum tumor markers and *BRCA1/2* germline mutations and analyze their associations with ovarian cancer.

**Methods:**

Levels of 11 serum tumor markers were examined in ovarian cancer patients and controls with benign gynecologic diseases. By integrating multiplex PCR and next‐generation sequencing technologies, *BRCA1/2* germline mutations were analyzed and confirmed by Sanger sequencing. The discriminative models with serum tumor markers and *BRCA1/2* mutation status were constructed for ovarian cancer detection and patient stratification.

**Results:**

Among 11 markers, six of them were significantly elevated and only beta‐human chorionic gonadotropin (β‐HCG) was significantly reduced in ovarian cancer patients. A total of 54 (23.3%) ovarian cancer patients were found to harbor *BRCA1/2* deleterious mutations, and *BRCA1/2* mutations were significantly associated with Hereditary Breast and Ovarian Cancer‐related tumors and family history of cancer. Carbohydrate antigen 125 showed a good performance in ovarian cancer detection as a single marker (AUC = 0.799), while a panel of eight markers showed a good performance in BRCA1 mutation detection with an AUC value of 0.974. In addition, a panel of five serum tumor markers combined with *BRCA1/2* mutation status showed a good performance in lymph node metastasis prediction (AUC = 0.843).

**Conclusions:**

We found the association between *BRCA1/2* germline mutation status and serum tumor marker levels, and identified discriminative models that combined serum tumor markers with *BRCA1/2* mutation status for ovarian cancer detection and patient stratification.

## 1 INTRODUCTION

Ovarian cancer is the third most commonly diagnosed cancer and the first leading cause of the gynecologic malignancies related deaths, with 238,700 new cases and 151,900 deaths worldwide in 2012 (Lu & Chen, 2014; Torre et al., 2015). The incidence of ovarian cancer in China has increased annually during the past 10 years, with up to 52,100 new cases and 22,500 deaths in 2015 (Chen et al., 2016). Ovarian cancer is a heterogeneous disease that can originate from surface epithelial cells, specialized stromal cells or germ cells, which can be subdivided into several histological subtypes including serous, mucinous, endometrioid, and clear cell tumors (Weiderpass & Tyczynski, 2015). In addition, ovarian cancer is one of the most lethal gynecologic malignancies to diagnose and difficult to detect at early stage (Buzolin et al., 2017).

Serum tumor markers play a crucial role in ovarian cancer diagnosis. Carbohydrate antigen 125 (CA125), a transmembrane glycoprotein produced by coelomic epithelium, is the first recommended serum marker for early detection and monitoring relapse of ovarian cancer (Fan et al., 2016). The expression of CA125 can be used to evaluate the treatment of ovarian cancer, which has been considered to be the most helpful clinical serological marker of ovarian cancer, especially in serous carcinoma (Esselen et al., 2016). Human epididymis protein 4 (HE4) is a protein that consists of a single peptide and two whey acidic protein domains containing a “four‐disulfide core” with eight cysteine residues (Clauss, Lilja, & Lundwall, 2002). CA125 and HE4 have been approved by the FDA for monitoring treatment and detecting of ovarian cancer (Montagnana et al., 2011). Besides CA125 and HE4, many other serum markers have been studied in ovarian cancer. For example, carbohydrate antigen 15‐3 (CA15‐3) was over‐expressed in a wide variety of ovarian cancer (Williams et al., 2014) and elevated in approximately 70% of ovarian cancer patients, predominantly in those with advanced disease (Jeschke et al., 2012). The level of serum β‐HCG was associated with ovarian cancer, with high levels in advanced FIGO stages (III and IV), regardless of histological type of tumor (Djurdjevic, Maksimovic, Pantelic, Golubovic, & Curcic, 2011). These markers in ovarian cancer detection have been individually investigated in many laboratories and clinical tests. However, the sensitivity and specificity of these markers still need further evaluation in Chinese patients, and it is meaningful to find tumor marker panels with high sensitivity and specificity in ovarian cancer diagnosis.

*BRCA1* and *BRCA2 *are two high‐susceptibility genes for familial ovarian cancer (Miki et al., 1994; Wooster et al., 1995). Individuals who inherit *BRCA1/2 *germline mutations showed a high lifetime risk and early onset of ovarian cancer (Choi et al., 2015). Genetic testing for *BRCA1/2* mutations has been proved to be a key step in the risk assessment, prognosis, treatment, and prevention of ovarian cancer (Pan & Xie, 2017). Recently, screening* BRCA1/2 *mutations has been applied as a companion diagnostic test guiding clinical medication for ovarian cancer patients (Daly et al., 2017). Poly ADP‐ribose polymerase (PARP) inhibitors are novel targeted drugs, which have recently been approved to treat advanced ovarian cancer patients carrying *BRCA1/2 *mutations (Shi et al., 2017). Ovarian cancer patients with *BRCA1/2* mutations will most likely benefit from PARP inhibitors compared to non‐*BRCA* mutated patients (Dizon, 2017). *BRCA1/2* mutation detection could become a routine clinical practice for evaluation of women with ovarian cancer for personalized medicine (Spriggs & Longo, 2016).

To investigate serum tumor markers and *BRCA1/2* germline mutations in Chinese ovarian cancer patients, we examined the concentrations of serum tumor markers in 232 ovarian cancer patients and 219 controls with benign gynecologic diseases and screened the entire coding exons and exon‐intron boundaries of *BRCA1/2 *in ovarian cancer patients. The discriminative models with serum tumor markers and *BRCA1/2* mutation status were also established for ovarian cancer detection and patient stratification.

## 2 MATERIALS AND METHODS

### 2.1 Study subjects

The study was approved by the Ethics Committee of Hunan Cancer Hospital, Changsha, China. All participants had provided written informed consent for participation in this study. Preoperative peripheral blood samples were collected from 232 ovarian cancer patients and 219 controls with benign gynecologic diseases in Hunan Cancer Hospital from 2015 to 2017. The 219 female controls were diagnosed with benign gynecologic diseases including uterine fibroids, ovarian cyst, ovarian benign mass, ovary teratoma, endometriosis. The clinicopathological characteristics of all ovarian cancer patients were summarized in Table 1, and that of benign controls were summarized Table S2. All ovarian cancer patients were diagnosed pathologically by experienced gynecologic pathologists.

**Table 1:** The clinicopathological characteristics and their associations with *BRCA1/2* deleterious mutations in 232 ovarian cancer patients

Characteristics		No. (%)	*BRCA1* No. (%)	*BRCA2* No. (%)	*BRCA1/2* No. (%)	Non‐BRCA No. (%)	*P* a	*P* b	*P* c	*P* d
Age at diagnosis	≤40	27(11.64%)	3(7.90%)	0(0.00%)	3(5.56%)	24(13.48%)				
	41–50	85(36.64%)	15(39.47%)	5(31.25%)	20(37.04%)	65(36.52%)				
	51–60	71(30.60%)	16(42.11%)	5(31.25%)	21(38.89%)	50(28.09%)				
	≥61	49(21.12%)	4(10.53%)	6(37.50%)	10(18.52%)	39(21.91%)				
	Mean	51.11	51.21	55.38	52.44	50.71	0.093	0.990	0.088	0.390
Histological subtype							0.360	0.065	0.237	**0.011**
	Serous	176(75.86%)	34(89.47%)	14(87.50%)	48(88.89%)	128(71.91%)				
	Mucinous	16(6.90%)	0(0.00%)	0(0.00%)	0(0.00%)	16(8.99%)				
	Endometrioid	7(3.02%)	1(2.63%)	1(6.25%)	2(3.70%)	5(2.81%)				
	Clear cell	7(3.02%)	0(0.00%)	1(6.25%)	1(1.85%)	6(3.37%)				
	Others	21(9.05%)	1(2.63%)	0(0.00%)	1(1.85%)	20(11.24%)				
	Unknown	5(2.16%)	2(5.26%)	0(0.00%)	2(3.70%)	3(1.69%)				
FIGO stage^e^							0.651	**0.019**	0.282	**0.007**
	Ⅰ	48(20.69%)	1(2.63%)	1(6.25%)	2(3.70%)	46(25.84%)				
	II	22(9.48%)	3(7.89%)	2(12.50%)	5(9.26%)	17(9.55%)				
	III	118(50.86%)	24(63.16%)	8(50.00%)	32(59.26%)	86(48.31%)				
	IV	25(10.78%)	5(13.16%)	3(18.75%)	8(14.81%)	17(9.55%)				
	Unknown	19(8.19%)	5(13.16%)	2(12.50%)	7(12.96%)	12(6.74%)				
Grade							0.944	0.335	0.647	0.256
	Low	11(4.74%)	3(7.89%)	1(6.25%)	4(7.41%)	7(3.93%)				
	Middle	22(9.48%)	2(5.26%)	1(6.25%)	3(5.56%)	19(10.67%)				
	High	134(57.76%)	26(68.42%)	13(81.25%)	39(72.22%)	95(53.37%)				
	Unknown	65(28.02%)	7(18.42%)	1(6.25%)	8(14.81%)	57(32.02%)				
Lymph node metastasis^f^							0.594	**0.025**	0.354	**0.024**
	Positive (+)	98(42.24%)	22(57.89%)	8(50.00%)	30(55.56%)	68(38.20%)				
	Negative (−)	134(57.76%)	16(42.11%)	8(50.00%)	24(44.44%)	110(61.80%)				
Menstrual age							0.437	0.576	0.580	0.707
	≤13	72(31.03%)	13(34.21%)	6(37.50%)	19(35.19%)	53(29.78%)				
	14–15	100(43.10%)	13(34.21%)	8(50.00%)	21(38.89%)	79(44.38%)				
	≥16	53(22.84%)	10(26.32%)	2(12.50%)	12(22.22%)	41(23.03%)				
	Unknown	7(3.02%)	2(5.26%)	0(0.00%)	2(3.70%)	5(2.81%)				
Menopausal age							0.314	0.389	0.354	0.447
	Premenopausal	70(30.17%)	9(23.68%)	4(25.00%)	13(24.07%)	57(32.02%)				
	≤45	41(17.67%)	10(26.32%)	1(6.25%)	11(20.37%)	30(16.85%)				
	46–50	67(28.88%)	11(28.95%)	8(50.00%)	19(35.19%)	48(26.97%)				
	≥51	43(18.53%)	5(13.16%)	3(18.75%)	8(14.81%)	35(19.66%)				
	Unknown	11(4.74%)	3(7.89%)	0(0.00%)	3(5.56%)	8(4.49%)				
Parity							0.321	0.834	0.185	0.475
	≤1	31(13.36%)	4(10.53%)	1(6.25%)	5(9.26%)	26(14.61%)				
	2–3	120(51.72%)	19(50.00%)	12(75.00%)	31(57.41%)	89(50.00%)				
	≥4	75(32.33%)	13(34.21%)	3(18.75%)	16(29.63%)	59(33.15%)				
	Unknown	6(2.59%)	2(5.26%)	0(0.00%)	2(3.70%)	4(2.25%)				
Personal history of cancer							0.313	0.139	1.000	0.418
	Yes	14(6.03%)	5(13.16%)	0(0.00%)	5(9.26%)	9(5.06%)				
	No	218(93.97%)	33(86.84%)	16(100.00%)	49(90.74%)	169(94.94%)				
HBOC^g^‐related tumor							0.894	**<0.001**	**0.004**	**<0.001**
	Yes	11(4.74%)	6(15.79%)	3(18.75%)	9(16.67%)	2(1.12%)				
	No	221(95.26%)	32(84.21%)	13(81.25%)	45(83.33%)	176(98.88%)				
Family history of tumors							0.811	**0.049**	0.377	**0.014**
	Yes	38(16.38%)	10(26.32%)	4(25.00%)	14(25.93%)	24(13.48%)				
	No	194(83.62%)	28(73.68%)	12(75.00%)	40(74.07%)	154(86.52%)				

Note

Here, *p* values for comparing difference of age were calculated by the Wilcoxon rank sum test; while *P* values for comparing categorical variables across other clinicopathological characteristics were calculated by χ^2^ test; *p* value < 0.05 in bold.

a *BRCA1* mutation carriers versus *BRCA2* mutation carriers.

b *BRCA1* mutation carriers versus non‐*BRCA* mutation carriers.

c *BRCA2* mutation carriers versus non‐*BRCA* mutation carriers.

d *BRCA1/2* mutation carriers versus non‐*BRCA* mutation carriers.

e FIGO: International Federation of Gynecology and Obstetrics.

f Lymph node metastasis was detected with histopathology during surgery.

g HBOC: Hereditary Breast and Ovarian Cancer.

### 2.2 Measurement of serum tumor markers

Levels of several serum tumor markers, including alpha‐fetoprotein (AFP), β‐HCG, CA125, CA15‐3, carbohydrate antigen 19‐9 (CA19‐9), carbohydrate antigen 242 (CA242), carcinoembryonic antigen (CEA), ferritin, human growth hormone (HGH), neuron‐specific enolase (NSE), were measured by Protein Chip‐Chemiluminescence (Health Digit, Huzhou, China). HE4 were measured separately with ELISA method. The measurement of HE4 level was only collected in 61 ovarian cancer patients and 60 controls due to the late clinical adoption of HE4 test started in year 2017 in this cohort.

### 2.3 Sequencing experiments and mutation analysis

Genomic DNA was extracted from each blood sample using TIANamp Genomic DNA Kit (TianGen Biotech, Beijing, China) and quantified using Nanodrop 2000 (Thermo Fisher Scientific, Wilmington, DE). Target enrichment and library preparation were performed by PCR using Human *BRCA1/2* Sequencing Panel Kit (Sansure Biotech, Hunan, China) according to the manufacture's instruction, which can amplify the entire coding exons and exon‐intron boundaries of *BRCA1/2* genes simultaneously. The library PCR products were purified with AMPure XP system (Beckman Coulter, Brea, CA), and quantified using Qubit^®^ dsDNA HS Assay Kit (Life Technologies, Foster City, CA) then pooled into one complete library with equimolar ratio. The prepared libraries were sequenced on MiSeq system (Illumina, San Diego, CA) using MiSeq Reagent Kit v2 (500 cycles). Analysis of sequencing data, mutation annotation, and mutation confirmation by Sanger sequencing were performed using our previously described methods (Li et al., 2017). The sequence numbering was based on transcript and protein sequence of *BRCA1 *(NM_007294.3 and NP_009225.1) and *BRCA2* (NM_000059.3 and NP_000050.2), respectively.

### 2.4 Statistical analysis

Continuous data were summarized using mean and standard deviation and the difference was determined by the *t* test. The χ^2^ test was used to compare categorical variables between groups across clinicopathological characteristics except age at diagnosis. Alternatively, Fisher's exact test was used when χ^2^ test was violated. The Wilcoxon rank sum test was used to analyze the difference of age at diagnosis and levels of serum tumor markers between *BRCA1/2 *mutation carriers and non‐*BRCA* mutation carriers. The obtained *p *values were considered statistically significant if the *p* value is <0.05. The false discovery rate procedure was used to adjust *p* values for multiple testing (Holm, 1979). Logistic regression analysis was used to construct discriminative models with serum tumor markers and *BRCA1/2* mutation status for ovarian cancer detection and patient stratification. The predictive performance of logistic regression models was evaluated by sensitivity, specificity, accuracy, and the area under the ROC curve (AUC) measurements. The leave‐one‐out cross‐validation prediction error was also estimated as a performance measurement for these models. All of the computations were performed using the R software (version 3.3.3, http://www.cran.r-project.org).

## 3 RESULTS

### 3.1 Levels of serum tumor markers in ovarian cancer and the associations with clinicopathological characteristics

**Figure 1:**
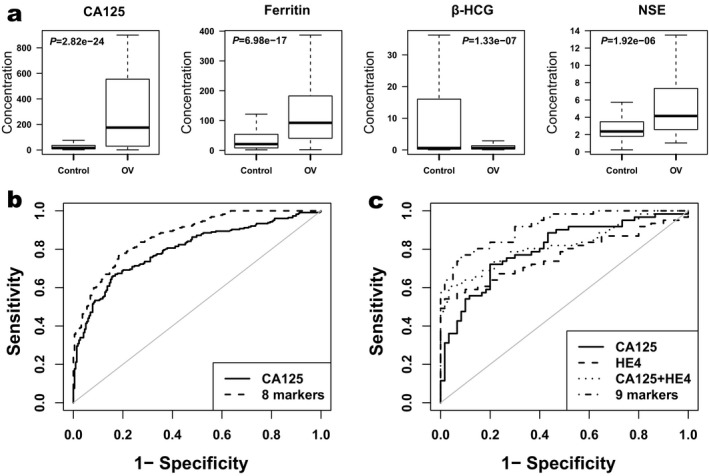
Serum levels of tumor markers and the ROC curve analysis of logistic regression models in ovarian cancer detection. (a) Boxplots show comparison of average serum levels of tumor markers (CA125, Ferritin, β‐HCG and NSE, respectively) between 232 ovarian cancer patients and 219 controls. *P* values were calculated using the *t* test and corrected by the false discovery rate (FDRs) correction procedure. (b) The receiver operating characteristic (ROC) curve analysis for ovarian cancer detection in 232 ovarian cancer patients and 219 controls. Here CA125 marked with solid line; the panel of eight markers (AFP, β‐HCG, CA125, CA15‐3, CEA, Ferritin, HGH, NSE) marked with dashed line. (c) The ROC curve analysis for individuals with HE4 levels in ovarian cancer detection. Here, CA125 marked with solid line; HE4 marked with dashed line; CA125 + HE4 marked with dotted line and the panel of nine markers (AFP, β‐HCG, CA125, CA15‐3, CEA, Ferritin, HE4, HGH, NSE) marked with dotdashed line.

Among the 11 serum tumor markers, AFP, CA125, CA19‐9, CA242, CEA, Ferritin, NSE and HE4 showed elevated levels and CA15‐3, HGH, and β‐HCG showed reduced levels in ovarian cancer patients (Table 2). The levels of CA125, CA19‐9, CEA, Ferritin, NSE, and HE4 were significantly elevated (*p* = 2.82E‐24, 0.021, 0.029, 6.98E‐17, 1.92E‐06, and 1.63E‐07, respectively) and the level ofβ‐HCG was significantly reduced (*p* = 1.33E‐07) in ovarian cancer patients. The serum levels of CA125, Ferritin, β‐HCG, and NSE were displayed in Figure 1a. The mean level of CA125 was elevated more than fivefold in ovarian cancer patients when comparing to that in controls (296.14 U/mL vs. 55.74 U/mL). In contrast, the mean level of β‐HCG was dramatically reduced in ovarian cancer patients when comparing to that in controls (0.9 ng/ml vs. 26.69 ng/ml). In addition, the mean level of HE4 was also elevated more than sixfold in ovarian cancer patients (271.29 ng/ml vs. 40.33 ng/ml), although it was measured only in 61 ovarian cancer patients and 60 controls.

**Table 2:** Comparison of serum tumor markers between ovarian cancer patients and benign controls

Tumor marker	Concentration (mean ± *SD*)^a^			Corrected *p *value^b^
	Benign controls	Ovarian cancer patients	Direction	
AFP (ng/mL)	3.28 ± 3.06	8.52 ± 37.67	↑	0.054
**CA125** (U/mL)	55.74 ± 115.51	296.14 ± 294.50	↑	**2.82E‐24**
CA15−3 (U/mL)	31.09 ± 99.33	19.64 ± 25.42	↓	0.11
**CA19−9 **(U/mL)	24.49 ± 63.80	46.83 ± 113.20	↑	**0.021**
CA242 (U/mL)	9.23 ± 30.45	14.35 ± 31.36	↑	0.101
**CEA **(ng/mL)	1.55 ± 5.52	3.52 ± 11.04	↑	**0.029**
**Ferritin **(ng/mL)	46.49 ± 71.19	128.93 ± 117.89	↑	**6.98E‐17**
HGH (ng/ml)	0.62 ± 2.65	0.44 ± 0.98	↓	0.329
**NSE** (ng/mL)	3.61 ± 5.08	6.59 ± 7.32	↑	**1.92E‐06**
**β‐HCG** (ng/mL)	26.69 ± 67.05	0.90 ± 0.81	↓	**1.33E‐07**
**HE4 **(pmol/L)^c^	40.33 ± 14.73	271.29 ± 304.22	↑	**1.63E‐07**

a SD: Standard deviation.

b *p* value calculated by the *t* test and corrected using FDR's correction procedure, and corrected *p* value <0.05 in bold.

c The measurement of HE4 level was only collected in 61 ovarian cancer patients and 60 benign controls.

In addition, CA19‐9 and Ferritin was significantly higher in elder and younger patients. CA125, CA15‐3, and Ferritin level was significantly associated with Histological subtype. CA125, Ferritin, NSE, and β‐HCG level was significantly associated with FIGO stage. In high grade tumor the CA125, CA15‐3, and Ferritin were significantly high expressed. Moreover, an association between Ferritin level and Menopausal age was found in ovarian cancer patients.

### 3.2 Deleterious germline mutations of *BRCA1/2* in ovarian cancer patients

**Figure 2:**
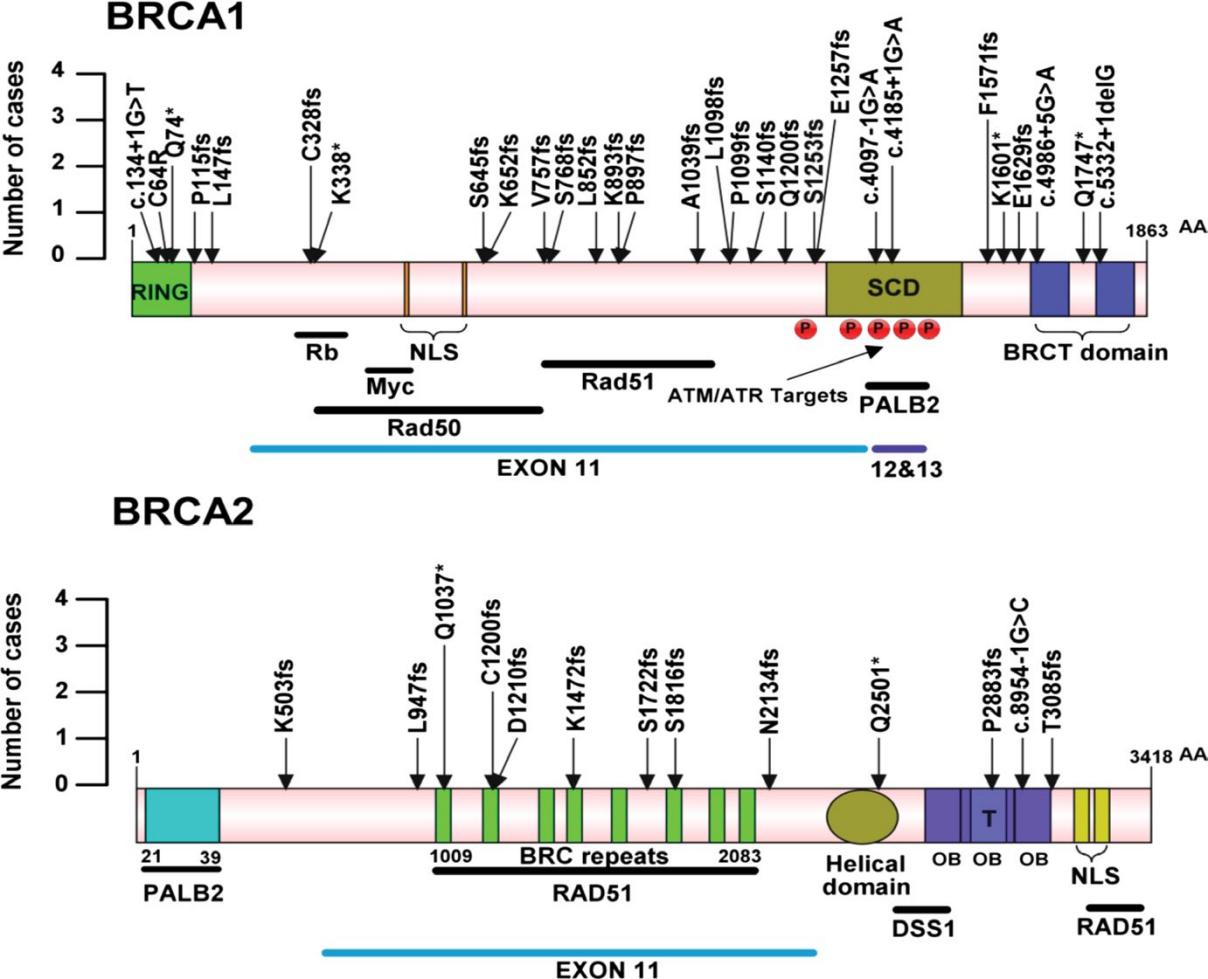
Schematic representation of *BRCA1/2* deleterious mutations in functional domains and protein binding regions in 232 ovarian cancer patients. Single nucleotide variants and small indels mapped to the *BRCA1/2* protein sequences are shown. Arrows point to amino acid mutation positions, and height of the arrows indicates the number of cases. Protein domains are shown as colored bars, RING: RING domain; NLS: nuclear localization sequence; SCD: serine containing domain; BRCT: *BRCA1* C Terminus; T: tower domain; OB: oligonucleotide/oligosaccharide binding. Horizontal solid black lines indicate protein binding domains for the listed binding partners. Red circles with letter P mark phosphorylation sites

Among the 232 ovarian cancer patients, a total of 43 deleterious germline mutation loci were identified in 54 ovarian cancer patients (Table 3). The entire frequency of *BRCA1/2* deleterious germline mutations in this study was 23.3% (54/232). Among these 43 mutations, there were 30 frameshift mutations (six insertions, 21 deletions, three deletion‐and‐insertions), six stop‐gain mutations, six splicing mutations, and one missense mutation. Except for the six splicing mutations, all of the *BRCA1/2 *mutations were illustrated on the protein structures in Figure 2. All deleterious mutations were confirmed by Sanger sequencing (Figure S1).

**Table 3:** Deleterious germline mutations of *BRCA1/2* found in 232 ovarian cancer patients

Gene	Exon	Nucleotide change^a^	Effect on protein^a^	dbSNP ID	Mutation type^b^	Previously reported^c^	#Cases	HBOC‐related Cases
*BRCA1*	Intron3	c.134+1G>T	—	rs80358043	Splicing	BIC|ClinVar	1	0
	exon5	c.190T>C	p.C64R	rs80357064	Missense	BIC|ClinVar|UMD|LOVD	1	0
	exon6	c.220C>T	p.Q74*	rs80357234	Stop‐gain	BIC|ClinVar|UMD	2	1
	exon7	c.342_343delTC	p.P115*	rs80357881	Frameshift del	BIC|ClinVar|UMD	1	0
	exon7	c.440delT	p.L147fs	.	Frameshift del	Novel	1	0
	exon11	c.981_982delAT	p.C328fs	rs80357772	Frameshift del	BIC|ClinVar|UMD|LOVD	2	0
	exon11	c.1012A>T	p.K338*	rs397508826	Stop‐gain	ClinVar	1	0
	exon11	c.1934delC	p.S645fs	.	Frameshift del	https://doi.org/10.18632/oncotarget.7027	1	0
	exon11	c.1952dupA	p.K652fs	rs80357885	Frameshift ins	BIC|ClinVar	1	0
	exon11	c.2269_2269delG	p.V757fs	rs80357583	Frameshift del	BIC|ClinVar|UMD|LOVD	1	0
	exon11	c.2302delA	p.S768fs	.	Frameshift del	Novel	1	0
	exon11	c.2553_2554insGAAAAGTGAA	p.L852fs	.	Frameshift ins	Novel	1	0
	exon11	c.2679_2682delGAAA	p.K893fs	rs80357596	Frameshift del	BIC|ClinVar|UMD|LOVD	1	0
	exon11	c.2685_2686delAA	p.P897fs	rs80357636	Frameshift del	BIC|ClinVar|UMD|LOVD	1	0
	exon11	c.3114delA	p.A1039fs	.	Frameshift del	Novel	1	1
	exon11	c.3288_3289delAA	p.L1098fs	rs80357686	Frameshift del	BIC|ClinVar|UMD	2	0
	exon11	c.3294delT	p.P1099fs	rs876658626	Frameshift del	ClinVar	1	0
	exon11	c.3418_3419insTGACTACT	p.S1140fs	.	Frameshift ins	https://doi.org/10.1007/s00432-017-2465-8	1	0
	exon11	c.3599_3600delAG	p.Q1200fs	rs398122674	Frameshift del	ClinVar	1	0
	exon11	c.3756_3759delGTCT	p.S1253fs	rs80357868	Frameshift del	BIC|ClinVar|UMD|LOVD	1	0
	exon11	c.3758_3759delCT	p.S1253fs	.	Frameshift del	https://doi.org/10.18632/oncotarget.10814	1	0
	exon11	c.3770_3771delAG	p.E1257fs	rs80357579	Frameshift del	BIC|ClinVar|UMD|LOVD	3	2
	Intron11	c.4097‐1G>A	—	rs80358070	Splicing	BIC|ClinVar|UMD|LOVD	1	0
	Intron11	c.4185+1G>A	—	rs80358076	Splicing	ClinVar|LOVD	2	0
	exon16	c.4712delT	p.F1571fs	rs886037790	Frameshift del	ClinVar	3	1
	exon16	c.4801A>T	p.K1601*	rs80357303	Stop‐gain	BIC|ClinVar	1	1
	exon16	c.4886_4887delinsC	p.E1629fs	.	Frameshift delins	Novel	1	0
	Intron16	c.4986+5G>A	—	rs397509211	Splicing	ClinVar	1	0
	exon20	c.5239C>T	p.Q1747*	rs80357367	Stop‐gain	BIC|ClinVar|LOVD	1	0
	Intron21	c.5332+1delG	—	rs397509263	Splicing	ClinVar|https://doi.org/10.1007/s10549_011_1596-x	1	0
*BRCA2*	exon10	c.1508_1509delinsT	p.K503fs	.	Frameshift delins	Novel	1	0
	exon11	c.2841_2849delinsTGTTCTCC	p.L947fs	.	Frameshift delins	https://doi.org/10.1038/modpathol.2016.135	1	0
	exon11	c.3109C>T	p.Q1037*	rs80358557	Stop‐gain	BIC|ClinVar|UMD	3	1
	exon11	c.3598_3599delTG	p.C1200fs	rs80359391	Frameshift del	BIC|ClinVar|LOVD	2	0
	exon11	c.3628_3629delGA	p.D1210fs	.	Frameshift del	Novel	1	1
	exon11	c.4415_4418delAGAA	p.K1472fs	rs397507333	Frameshift del	ClinVar	1	0
	exon11	c.5164_5165delAG	p.S1722fs	rs80359490	Frameshift del	BIC|ClinVar|LOVD	1	0
	exon11	c.5446dupA	p.S1816fs	.	Frameshift ins	Novel	1	0
	exon11	c.6400_6401delAA	p.N2134fs	.	Frameshift del	Novel	1	0
	exon15	c.7501C>T	p.Q2501*	.	Stop‐gain	UMD	1	0
	exon21	c.8645_8646dupAA	p.P2883fs	.	Frameshift ins	Novel	1	0
	Intron22	c.8954‐1G>C	—	.	Splicing	Novel	1	0
	exon24	c.9253dupA	p.T3085fs	rs80359752	Frameshift ins	BIC|ClinVar|UMD	1	1

a The sequence numbering was based on transcript and protein sequence of *BRCA1* (NM_007294.3 and NP_009225.1) and *BRCA2* (NM_000059.3 and NP_000050.2), respectively.

b SNV: single‐nucleotide variant; del: deletion; ins: insertion.

c Novel variants were defined as variants that have not been previously recorded in BIC (http://research.nhgri.nih.gov/bic/), UMD (http://www.umd.be/), NCBI ClinVar (http://www.ncbi.nlm.nih.gov/clinvar/), LOVD (http://databases.lovd.nl/shared/genes/), or COSMIC (http://cancer.sanger.ac.uk/cosmic/), nor reported in the literature.

For *BRCA1*, 30 deleterious germline mutations were found in 38 patients (38/232, 16.4%), including six recurrent mutations (c.3770_3771delAG and c.4712delT each present in 3 patients, c.220C>T, c.981_982delAT, c.3288_3289delAA, and c.4185+1G>A each in 2 patients) and five novel mutations (c.440delT, c.2302delA, c.2553_2554insGAAAAGTGAA, c.3114delA, and c.4886_4887delinsC). For *BRCA2, *13 deleterious germline mutations were found in 16 (6.9%) patients, including two recurrent mutations (c.3109C>T in 3 cases and c.3598_3599delTG in 2 cases). A total of six mutations in *BRCA2* (c.1508_1509delinsT, c.3628_3629delGA, c.5446dupA, c.6400_6401delAA, c.8645_8646dupAA and c.8954‐1G>C) were novel.

### 3.3 Associations of *BRCA1/2* mutations with clinicopathological characteristics and serum tumor markers

The associations of *BRCA1/2 *deleterious germline mutation status with clinicopathological characteristics of the 232 patients were summarized in Table 1. Regarding to histological subtype of ovarian cancer patients, there was statistically significant difference between *BRCA1/2 *mutation carriers and non‐*BRCA* mutation carriers in histological subtype (*p = *0.011). In addition, the FIGO stage of *BRCA1* mutation carriers and *BRCA1/2 *mutation carriers were significantly different from non‐*BRCA* mutation carriers (*p = *0.019 and 0.007 respectively). Notably, *BRCA1* mutation carriers and *BRCA1/2* mutation carriers were significantly more likely to be positive in lymph node metastasis, when compared with non‐*BRCA* mutation carriers (*p = *0.025 and 0.024, respectively).

We also investigated whether deleterious germline mutations were associated with Hereditary Breast and Ovarian Cancer (HBOC) related tumor (Table 1). In this study, 38 patients showed family history of cancer and 11 patients had HBOC‐related tumors. Overall, 9 in 11 patients with HBOC‐related tumor had *BRCA1/2 *deleterious mutations. Compared to non‐*BRCA* mutation carriers, *BRCA1*, *BRCA2*, and *BRCA1/2* mutations carriers all showed significant association with HBOC‐related tumors (*p < *0.001, *p = *0.004, and *p < *0.001, respectively). *BRCA1* mutation carriers and *BRCA1/2* mutation carriers also showed significant association with family history of cancer, when compared with non‐*BRCA* mutation carriers (*p = *0.049 and 0.014, respectively).

The associations between *BRCA1/2* deleterious germline mutation status and serum levels of tumor markers were summarized in Table S1. The levels of CA125 in *BRCA1* and *BRCA1/2* mutation carriers were significantly higher than that in non‐*BRCA *mutation carriers (*p = *0.010 and 0.040, respectively). *BRCA1* mutation carriers had significantly higher levels of CA15‐3, β‐HCG (*p = *0.035 and 0.027 respectively), and significantly lower level of HGH (*p = *0.027) than non‐*BRCA *mutation carriers. No significant difference was found in other serum tumor markers.

### 3.4 Logistic regression analysis for ovarian cancer detection and patient stratification

By logistic regression analysis, we evaluated the performance of tumor markers in ovarian cancer detection and patient stratification (Table 4). Among the 10 serum tumor markers in 232 patients and 219 controls, CA125 showed the best performance in ovarian cancer detection as a single marker (AUC = 0.799, Sensitivity = 0.551, Specificity = 0.881) (Table 4, Figure 1b). In the combinations of different markers, a panel of eight markers (AFP, β‐HCG, CA125, CA15‐3, CEA, Ferritin, HGH, and NSE) showed the best performance in ovarian cancer detection (AUC = 0.873, Sensitivity = 0.727, Specificity = 0.826) (Table 4, Figure 1b). Although we had limited number of samples with HE4 information, we investigated if adding this marker could improve detection performance. While only considering 61 ovarian cancer patients and 60 controls with HE4 serum levels, HE4 as a single marker showed a comparable performance in ovarian cancer detection (AUC = 0.767, Sensitivity = 0.541, Specificity = 0.933) to that of CA125 (AUC = 0.802, Sensitivity = 0.541, Specificity = 0.883), and the previous 8‐marker panel plus HE4 showed an improved performance in ovarian cancer detection (AUC = 0.916, Sensitivity = 0.770, Specificity = 0.900) (Table 4, Figure 1c). In patient stratification analysis, a panel of seven markers (β‐HCG, CA125, CA15‐3, CA19‐9, CA242, CEA, and HE4) showed a good performance in prediction of *BRCA1/2* mutation carriers (AUC = 0.881, Sensitivity = 0.583, Specificity = 0.939) (Table 4), and we analyzed the performance by integrating tumor markers with clinical characteristics in patient stratification. If adding lymph node metastasis as a parameter, the performance could be further improved (AUC = 0.917, Sensitivity = 0.667, Specificity = 0.980). Specially, a panel of eight serum tumor markers (AFP, β‐HCG, CA125, CA15‐3, CA19‐9, CA242, CEA, and HE4) showed a good performance in prediction of *BRCA1* mutation carriers (AUC = 0.974, Sensitivity = 0.714, Specificity = 1.000). In addition, a panel with *BRCA1* mutation, *BRCA2* mutation, and five serum markers (AFP, CA125, CA19‐9, CA242 and HE4) showed a good performance for identifying patients with lymph node metastasis (AUC = 0.843, Sensitivity = 0.600, Specificity = 0.902) (Table 4).

**Table 4:** The predictive performance of logistics regression models for classification

Classification (class distribution)	Model variables	CV error^a^	Sensitivity	Specificity	Accuracy	AUC
Ovarian cancer patients vs. controls (232, 219)^b^	CA125	0.189	0.551	0.881	0.713	0.799
	AFP, β‐HCG, CA125, CA15‐3, CEA, Ferritin, HGH, NSE	0.152	0.727	0.826	0.776	0.873
Ovarian cancer patients vs. controls with HE4 (61, 60)^c^	CA125	0.197	0.541	0.883	0.711	0.802
	HE4	0.178	0.541	0.933	0.736	0.767
	CA125, HE4	0.167	0.623	0.933	0.777	0.823
	AFP, β‐HCG, CA125, CA15‐3, CEA, Ferritin, HE4, HGH, NSE	0.152	0.770	0.900	0.835	0.916
*BRCA1/2* mutation carriers vs. non‐*BRCA* carriers (12, 49)^d^	β‐HCG, CA125, CA15‐3, CA19‐9, CA242, CEA, HE4	0.177	0.583	0.939	0.869	0.881
	lymph node metastasis, β‐HCG, CA125, CA15‐3, CA19‐9, CA242, CEA, HE4	0.154	0.667	0.980	0.918	0.917
*BRCA1* mutation carriers vs. non‐*BRCA1* carriers (7, 54)^d^	AFP, β‐HCG, CA125, CA15‐3, CA19‐9, CA242, CEA, HE4	0.152	0.714	1.000	0.967	0.974
	lymph node metastasis, AFP, β‐HCG, CA125, CA15‐3, CA19‐9, CA242, CEA, HE4	0.148	0.714	0.981	0.951	0.976
Lymph node metastasis: positive vs. negative (20, 41)^d^	AFP, CA125, CA19‐9, CA242, HE4	0.256	0.200	0.878	0.656	0.735
	*BRCA1* mutation, *BRCA2* mutation, AFP, CA125, CA19‐9, CA242, HE4	0.242	0.600	0.902	0.803	0.843

a The adjusted estimate of leave‐one‐out cross‐validation (LOOCV) prediction error.

b The models were based on 232 ovarian cancer patients and 219 controls.

c The models were based on 61 ovarian cancer patients and 60 controls with HE4 levels.

d The models were based on 61 ovarian cancer patients with HE4 levels.

## 4 DISCUSSION

Serum tumor markers and *BRCA1/2* germline mutations are crucial factors in cancer diagnosis, treatment, and prognosis. In this study, we comprehensively investigated multiple serum tumor markers and *BRCA1/2 *germline mutations in a Chinese cohort of ovarian cancer patients. The controls we used in this study were from women who visited hospital with benign gynecologic diseases, which might represent real clinic situation better than using healthy woman as control. We found that several serum tumor markers were associated with *BRCA1/2 *mutation status. *BRCA1/2* mutation status could improve serum tumor markers performance in ovarian cancer discriminative models.

*BRCA1/2* mutation status provides important information for the identification of patients that are most likely to benefit from treatment with PARP inhibitors and guides treatment decisions. In this study, the overall frequency of *BRCA1/2* deleterious germline mutation was 23.3% (16.38% in *BRCA1* and 6.90% in *BRCA2*) in patients from Hunan province in mid‐south China. This frequency was close to two multicenter studies from eastern and north China ovarian cancer patients by NGS (Shi et al., 2017; Wu et al., 2017) 16.7% (13.1% in *BRCA1* and 3.9% in *BRCA2*) and 28.4% (20.8% in *BRCA1* and 7.6% in *BRCA2*), respectively. HBOC is syndrome primarily associated with mutations in *BRCA1* or *BRCA2* genes. Same as previous study, the *BRCA1/2* germline mutations were significantly associated with HBOC‐related tumor or family history (Bolton et al., 2012; Maistro et al., 2016; Shi et al., 2017). These ovarian cancer patients carrying *BRCA1/2 *mutations could benefit from PARP inhibitors for targeted treatment (Shi et al., 2017).

Serum tumor markers play a crucial role in ovarian cancer diagnosis, prognosis prediction, and monitoring disease progression (Richards et al., 2015). CA125 as a single marker showed good performance in ovarian cancer detection (AUC = 0.799), but with low sensitivity in ovarian cancer detection model (Yang, Lu, & Bast, 2017). To overcome the limitation of single marker in ovarian cancer detection, combined detection of multi‐tumor markers has been suggested (Bian et al., 2014). In this study, we found a panel of eight serum tumor markers showing improved performance in ovarian cancer detection (AUC = 0.873) than single CA125 marker.

HE4 was another serum marker that has been approved by FDA. Overexpression of HE4 plays direct biological role in promoting ovarian cancer cell proliferation. In our ovarian cancer detection model, the prediction performance for ovarian cancer was improved with AUC value up to 0.916 when HE4 was included. In addition, the panels including HE4 could predict *BRCA1/2* mutation status, especially *BRCA1* mutation status with an AUC value as 0.974. A recent study reported that ovarian patients with *BRCA1* gene mutations have relatively low serum HE4 levels (Chudecka‐Glaz, Cymbaluk‐Ploska, Strojna, & Menkiszak, 2017). We also observed lower HE4 level in *BRCA1* mutation carriers than that in noncarriers, although the difference is not significant. The mechanism underneath this correlation is worth further investigation in the future. A limitation must be mentioned that HE4 detection was not included in the Protein Chip we used here, and the detection of HE4 was not applied in our hospital until 2017. HE4 level was only detected in 121 samples in our cohort and these results still need future validation in large cohorts.

It is very interesting that by adding lymph node metastasis status as a parameter in patient stratification, the prediction of *BRCA1/2* mutation status (AUC = 0.917) was much improved than only using serum markers (AUC = 0.881), and *BRCA1/2* mutation status together with five serum markers could predict lymph node metastasis status (AUC = 0.843) better than serum markers alone (AUC = 0.735). It has been reported that ovarian cancer patients with *BRCA1/2* mutations had significantly more bulky lymph nodes than patients with wild type *BRCA1/2* (Petrillo et al., 2017). Our results also indicated that *BRCA1/2* mutation carriers had more lymph node metastasis than nonmutation carriers, especially in *BRCA1* mutation carriers. Further investigation of the relationship between lymph node metastasis and *BRCA1/2* mutation status in ovarian cancer might provide more information for patient stratification.

In accordance with a previous study (Liu et al., 2017), we observed that the elevated serum level of CA125 was also associated with *BRCA1/2 *mutation. The serum level of HGH was significantly lower in BRCA1 mutation carriers than nonmutation carriers in our study. This may be related to proliferation mechanism that HGH levels in serum do not facilitate tumor cells proliferation (Santovena, Farina, Llabres, Zhu, & Dannies, 2010). Elevated serum level of CA15‐3, β‐HCG was significantly higher in BRCA1 mutation carriers than nonmutation carriers. Although the difference is not significant after adjusted, it still could be observed that there were differences in levels of CA15‐3, β‐HCG, HGH between BRCA1/2 mutation carriers, and non‐BRCA mutation carriers. Both genetic variations and serum tumor markers are crucial features of cancer. Combined genetics and serum tumor marker variation detection would improve cancer diagnosis and treatment in clinical. A recent research provided inspiring result by combining 12 tumor markers and 16 genes mutation analysis for cancer early detection including ovarian cancer (Cohen et al., 2018). The crucial tumor markers and mutations, such as AFP, CA125， CEA, CA199, HE4 and *TP53 *(OMIM:191170), *PIK3CA *(OMIM:171834), *EGFR *(OMIM:131550), *BRAF *(OMIM:164757) mutations were included in their cancer SEEK panel. The combined analysis of tumor markers and genetic variations would be a promising method for ovarian cancer detection and provide guideline for treatment. The panels we reported here could be alternative biomarkers for patient stratification using targeted therapy in ovarian cancer such as PARP inhibitor.

In conclusion, we analyzed serum tumor markers and the prevalence of *BRCA1/2 *germline mutations in Chinese ovarian cancer patients. The identified panels of serum tumor markers showed a good performance in ovarian cancer prediction. In addition, the serum tumor markers combining *BRCA1/2* mutation status could also predict metastasis status. These findings provided important information for ovarian cancer prediction and patient stratification, which eventually would benefit the diagnosis and treatment for ovarian cancer patients.

## ETHICAL STANDARDS

We declare that the experiments performed in this study comply with the current laws of the People's Republic of China.

## CONFLICT OF INTEREST

Ming Chen, Xinwu Guo, Xunxun Xu, Limin Peng, Lizhong Dai, and Jun Wang are employees of Sanway Gene Technology Inc. Julia X. Day is a student from La Jolla Country Day School (La Jolla, CA, USA), who worked as an intern at Sanway Gene Technology Inc.

## AUTHORS’ CONTRIBUTIONS

Hongyu Deng, Xunxun Xu, Guoli Li, Hui Jiang, JL, and Julia X. Day performed the experiments. Xinwu Guo, Ming Chen, and Limin Peng analyzed the data. Jun Wang, Lizhong Dai, and Xiaochun Wang conceived the study and participated in its design and coordination. Hongyu Deng, Dongyong Shan, Yinghua Li, Yanjie Zhou, and Bin Liu collected specimens for the project. Hongyu Deng, Xinwu Guo, Jianfu Heng, Ming Chen, and JW drafted the manuscript. All authors read and approved the final manuscript.
