# Supplementary material for: Comprehensive analysis of serum tumor markers and BRCA1/2 germline mutations in Chinese ovarian cancer patients
Source: Mol Genet Genomic Med. 2019 Apr 10;7(6):e672. doi: 10.1002/mgg3.672 (PMC6565576; doi:10.1002/mgg3.672)

Supporting Figure 1. Verification of deleterious germline mutations of *BRCA1/2* by Sanger sequencing

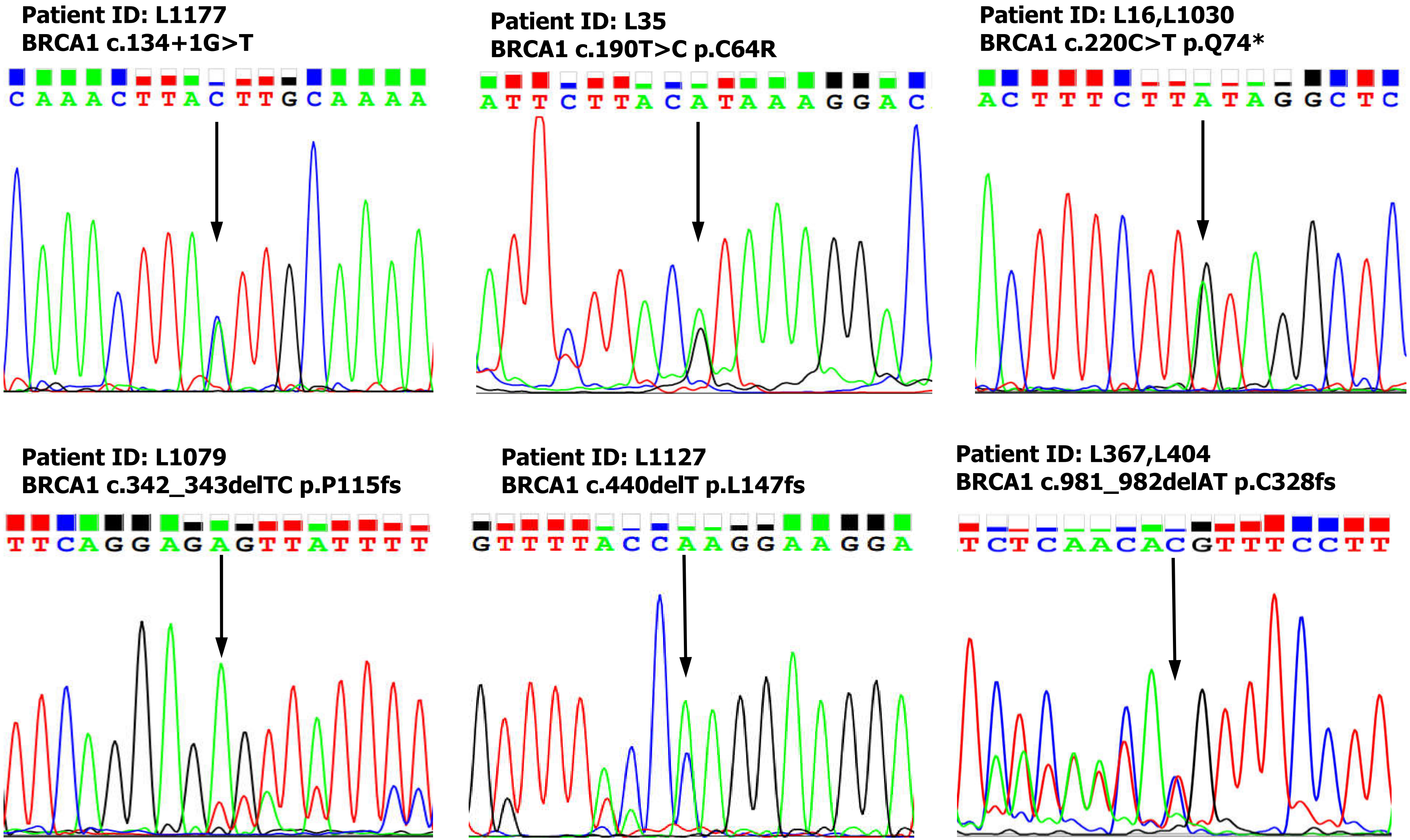

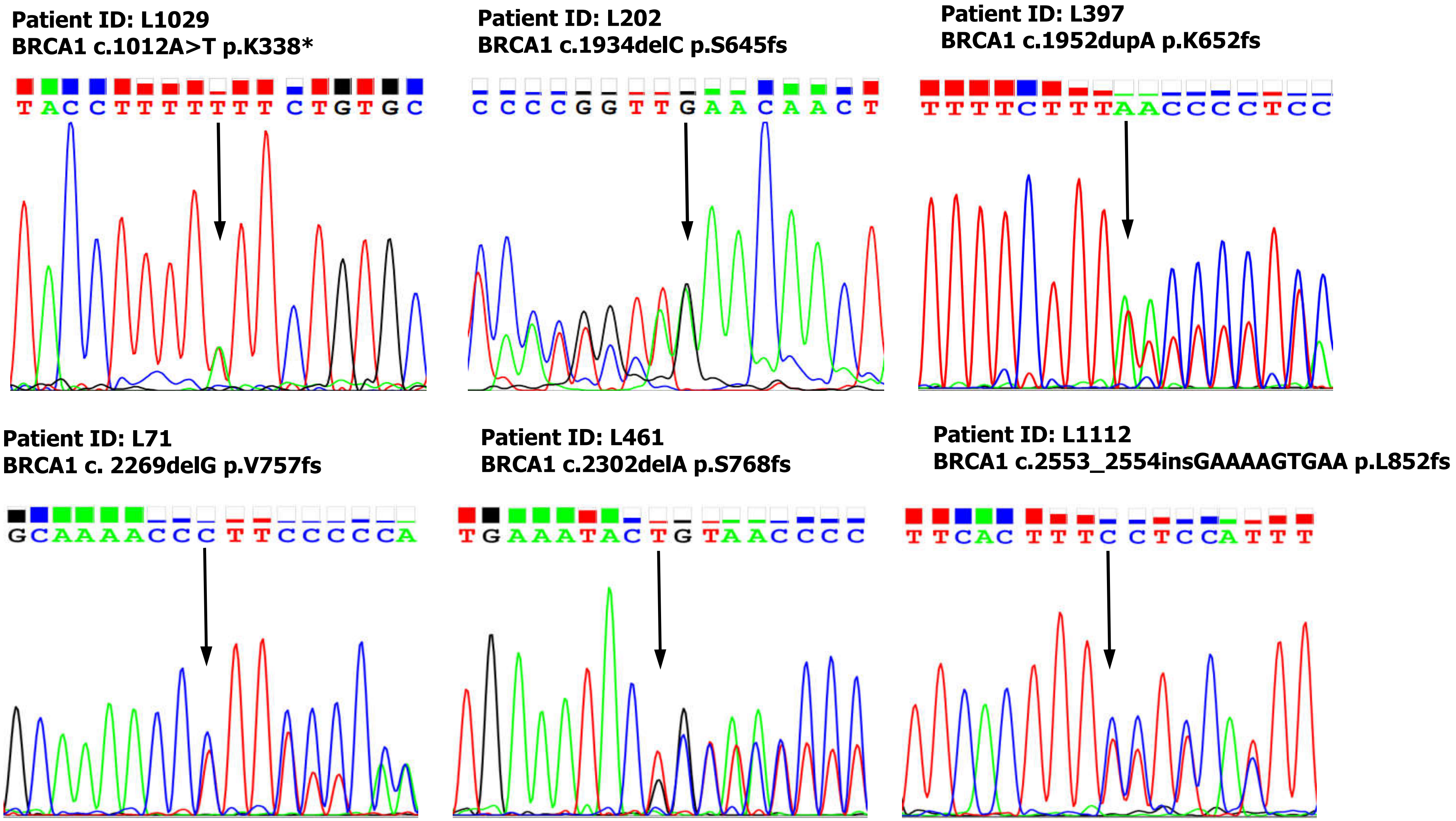
Patient ID: L1029
BRCA1 c.1012A>T p.K338*
TACCTTTTTTCTGTGC
Patient ID: L202
BRCA1 c.1934delC p.S645fs
CCCCGGTTGAACAACT
Patient ID: L397
BRCA1 c.1952dupA p.K652fs
TTTTCTTTAACCCCTCC
Patient ID: L71
BRCA1 c. 2269delG p.V757fs
GCAAAACCCTTCCCCCA
Patient ID: L461
BRCA1 c.2302delA p.S768fs
TGAAATACTGTAACCCC
Patient ID: L1112
BRCA1 c.2553_2554insGAAAAGTGAA p.L852fs
TTCACTTTCCTCCATTT

Patient ID: L1031
BRCA1 c.2679_2682delGAAA p.K893fs

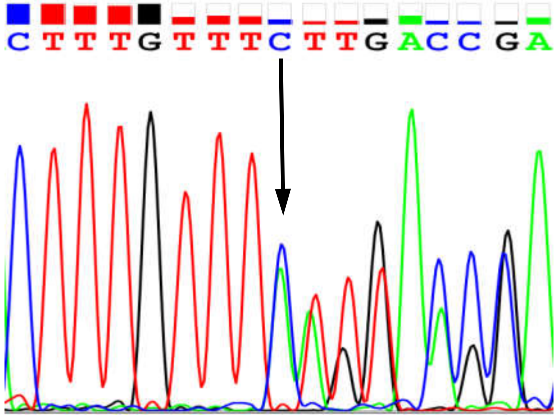

Patient ID: L72
BRCA1 c.2685_2686delAA p.P897fs

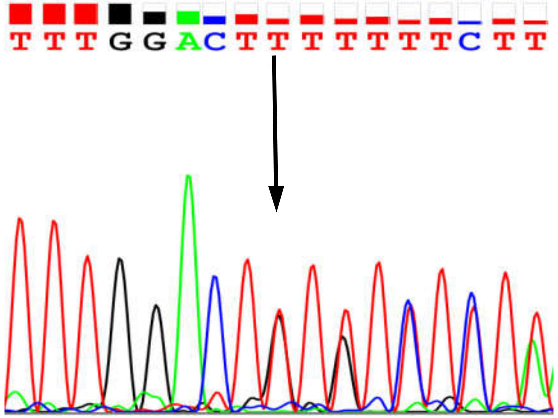

Patient ID: L1131
BRCA1 c.3114delA p.A1039fs

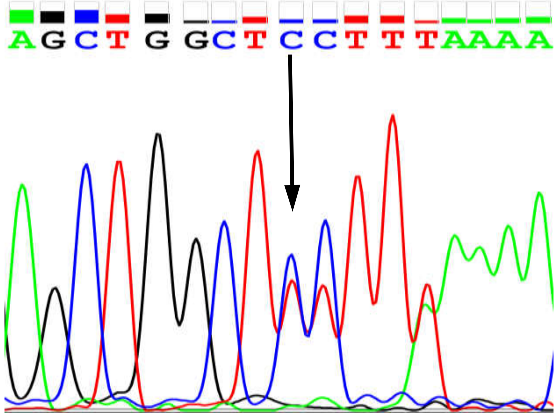

Patient ID: L237,L1129
BRCA1 c.3288_3289delAA p.L1098fs

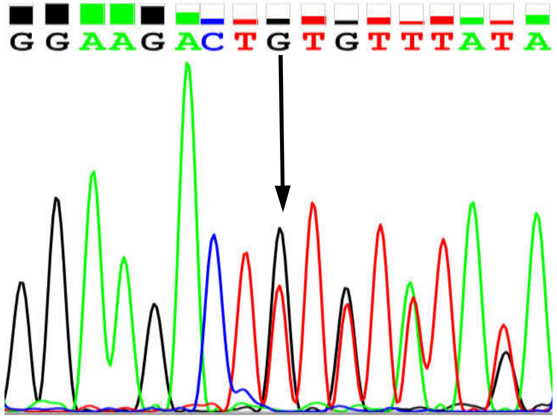

Patient ID: L238
BRCA1 c.3294delT p.P1099fs

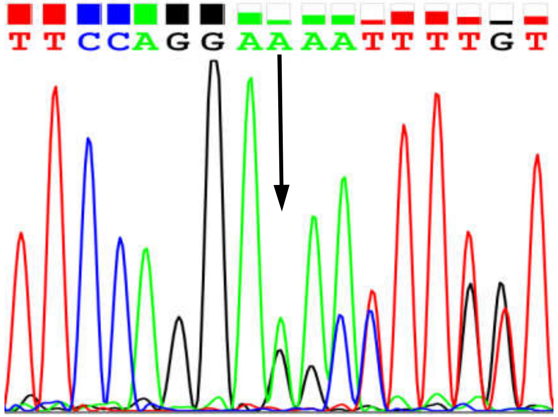

Patient ID: L287
BRCA1 c.3418_3419insTGACTACT p.S1140fs

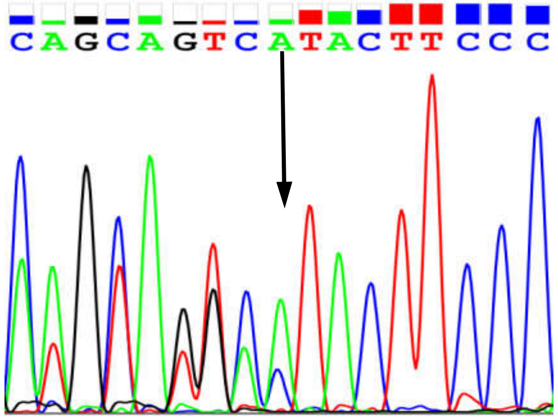

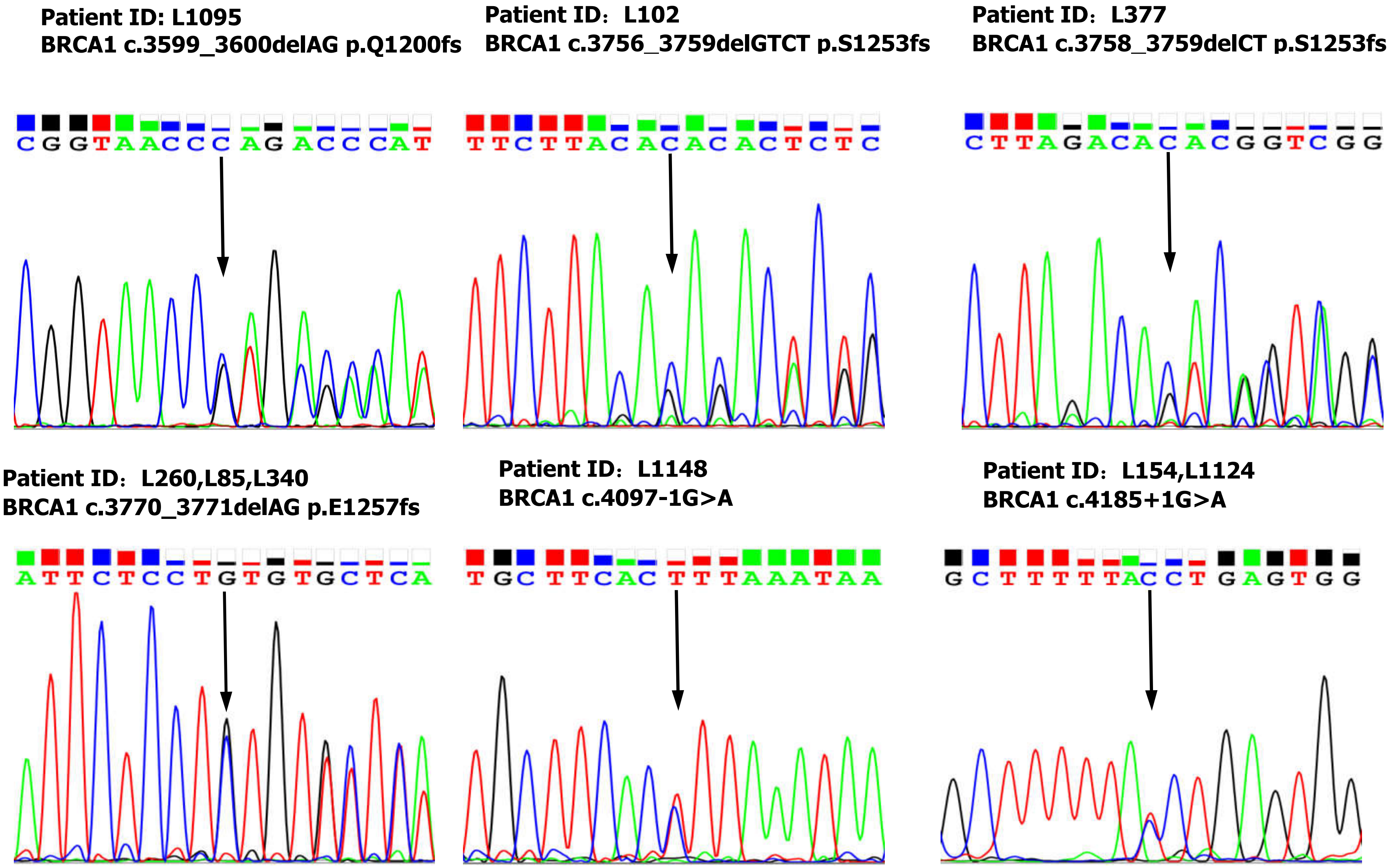

Patient ID: L1095
BRCA1 c.3599_3600delAG p.Q1200fs
CGGTAACCCAGACCCAT
Patient ID：L102
BRCA1 c.3756_3759delGTCT p.S1253fs
TTCTTACACACACTCTC
Patient ID：L377
BRCA1 c.3758_3759delCT p.S1253fs
CTTAGACACACGGTCGG
Patient ID：L260,L85,L340
BRCA1 c.3770_3771delAG p.E1257fs
ATTCTCCTGTGTGCTCA
Patient ID：L1148
BRCA1 c.4097-1G>A
TGCTTCACTTTAAATAA
Patient ID：L154,L1124
BRCA1 c.4185+1G>A
GCTTTTTACCTGAGTGG

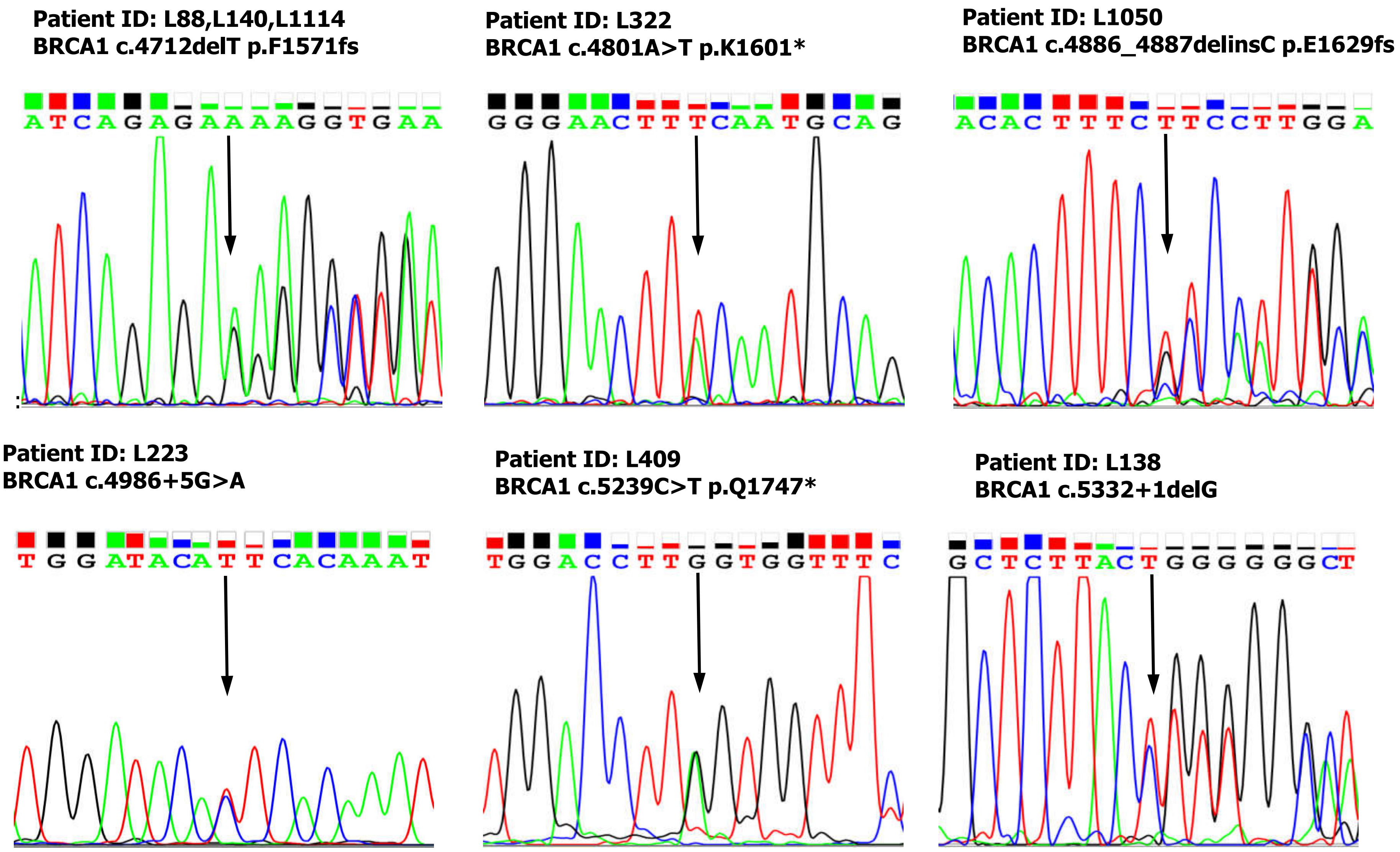
Patient ID: L88,L140,L1114
BRCA1 c.4712delT p.F1571fs
ATCAGAGAAAAGGTGAA
Patient ID: L322
BRCA1 c.4801A>T p.K1601*
GGGAACTTTCAATGCAG
Patient ID: L1050
BRCA1 c.4886_4887delinsC p.E1629fs
ACACTTTCTTCCTTGGA
Patient ID: L223
BRCA1 c.4986+5G>A
TGGATACATTCACAAAT
Patient ID: L409
BRCA1 c.5239C>T p.Q1747*
TGGACCTTGGTGGTTTC
Patient ID: L138
BRCA1 c.5332+1delG
GCTCTTACTGGGGGGCT

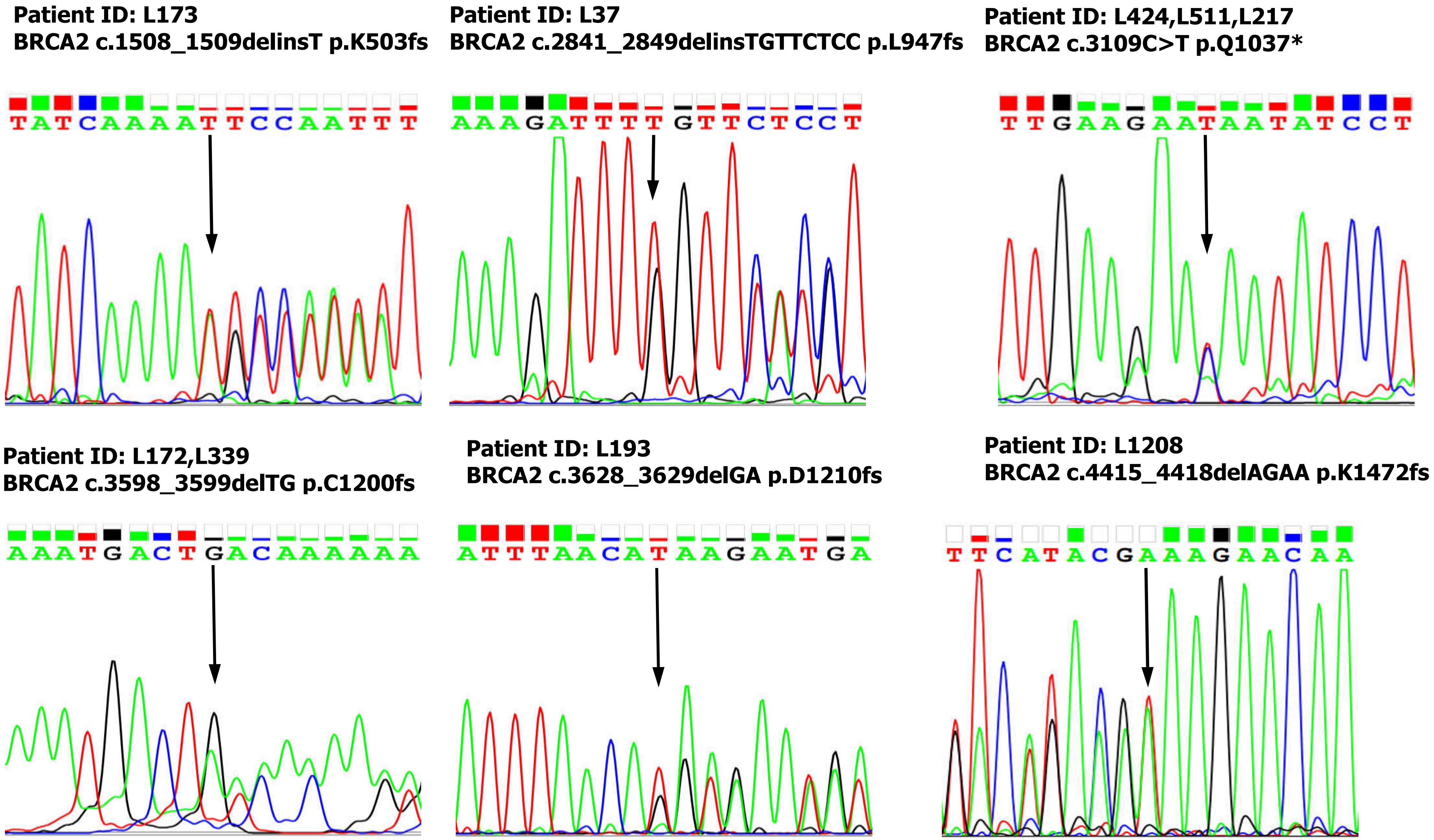
Patient ID: L173
BRCA2 c.1508_1509delinsT p.K503fs
Patient ID: L37
BRCA2 c.2841_2849delinsTGTTCTCC p.L947fs
Patient ID: L424,L511,L217
BRCA2 c.3109C>T p.Q1037*
Patient ID: L172,L339
BRCA2 c.3598_3599delTG p.C1200fs
Patient ID: L193
BRCA2 c.3628_3629delGA p.D1210fs
Patient ID: L1208
BRCA2 c.4415_4418delAGAA p.K1472fs

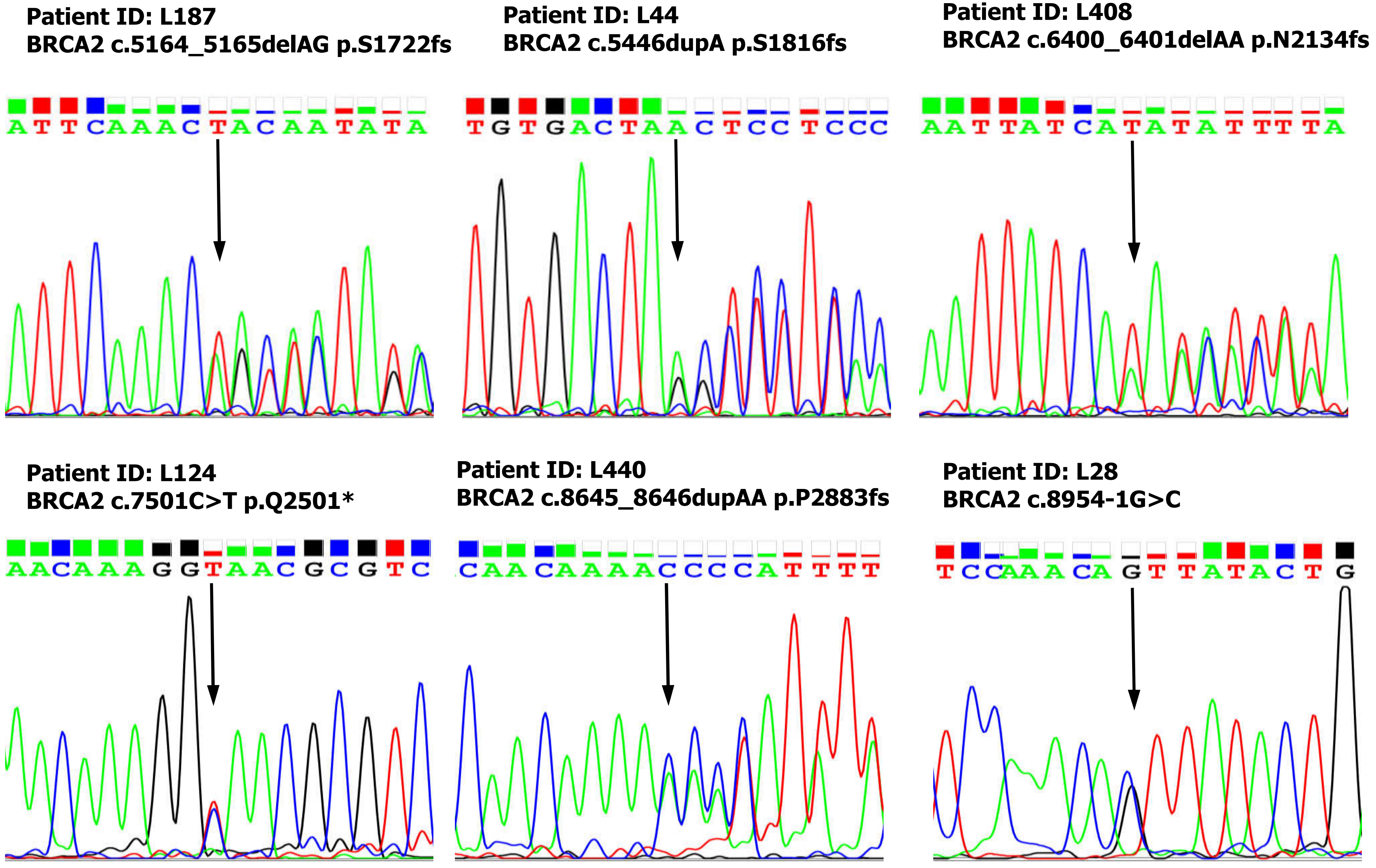

Patient ID: L187
BRCA2 c.5164_5165delAG p.S1722fs
ATTCAAACTACAATATA
Patient ID: L44
BRCA2 c.5446dupA p.S1816fs
TGTGACTAACTCCTCCC
Patient ID: L408
BRCA2 c.6400_6401delAA p.N2134fs
AATTATCATATATTTTA
Patient ID: L124
BRCA2 c.7501C>T p.Q2501*
AACAAAGGTAACGCGTC
Patient ID: L440
BRCA2 c.8645_8646dupAA p.P2883fs
CAACAAAACCCCATTTT
Patient ID: L28
BRCA2 c.8954-1G>C
TCCAAACAGTTATACTG

Patient ID: L1116
BRCA2 c.9253dupA p.T3085fs

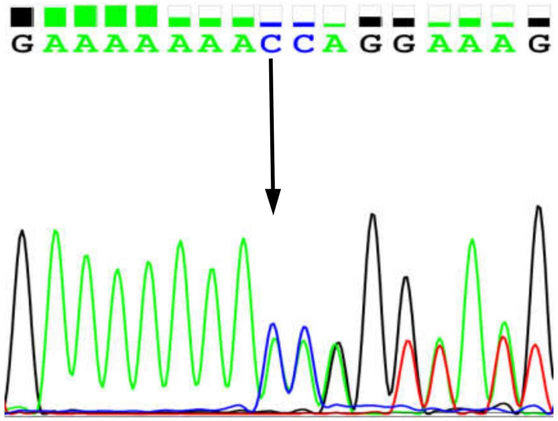

Supplement: Supplementary file 1 [file MGG3-7-e672-s001.pdf]
