# Supplementary material for: Comprehensive analysis of serum tumor markers and BRCA1/2 germline mutations in Chinese ovarian cancer patients
Source: Mol Genet Genomic Med. 2019 Apr 10;7(6):e672. doi: 10.1002/mgg3.672 (PMC6565576; doi:10.1002/mgg3.672)
Supplement: Supplementary file 2 [file MGG3-7-e672-s002.doc]

**Supporting Table 1. The associations of *BRCA* deleterious mutations with serum tumor markers in 232 ovarian cancer patients**

| **Tumor marker level** | ***BRCA1* (Mean±SD)** | ***BRCA2* (Mean±SD)** | ***BRCA1/2* (Mean±SD)** | **non-*BRCA* (Mean±SD)** | ***P* ^a^** | ***P* ^b^** | ***P* ^c^** | ***P* ^d^** |
| --- | --- | --- | --- | --- | --- | --- | --- | --- |
| AFP(ng/mL) | 3.03 ± 4.60 | 1.65 ± 1.16 | 2.61 ± 3.93 | 10.33 ± 42.84 | 0.846 | 0.797 | 0.884 | 0.734 |
| CA125(U/mL) | 426.56 ± 295.29 | 259.90 ± 229.40 | 376.25 ± 285.51 | 271.73 ± 293.66 | 0.28 | **0.01** | 0.884 | **0.04** |
| CA15-3(U/mL) | 25.68 ± 22.23 | 17.13± 16.76 | 23.1 ± 20.95 | 18.59 ± 26.6 | 0.439 | **0.035** | 0.884 | 0.09 |
| CA19-9(U/mL) | 50.12 ± 122.33 | 9.58 ± 7.43 | 37.88 ± 103.58 | 49.55 ± 116.11 | 0.631 | 0.585 | 0.65 | 0.294 |
| CA242(U/mL) | 14.32 ± 23.48 | 5.21 ± 2.78 | 11.57 ± 20.04 | 15.19 ± 34.08 | 0.396 | 0.585 | 0.884 | 0.747 |
| CEA(ng/mL) | 5.54 ± 20.19 | 1.31 ± 0.86 | 4.26 ± 16.92 | 3.29 ± 8.54 | 0.376 | 0.585 | 0.884 | 0.747 |
| Ferritin(ng/mL) | 114.03 ± 91.35 | 161.40 ± 115.43 | 128.33 ± 100.51 | 129.11 ± 122.96 | 0.363 | 0.992 | 0.65 | 0.63 |
| HGH(ng/mL) | 0.26 ± 0.61 | 0.53± 1.32 | 0.34 ± 0.88 | 0.47 ± 1.01 | 0.28 | **0.027** | 0.899 | 0.09 |
| NSE(ng/mL) | 8.51 ± 8.30 | 5.11 ± 3.37 | 7.48 ± 7.31 | 6.32 ± 7.32 | 0.396 | 0.222 | 0.899 | 0.43 |
| β-HCG(ng/mL) | 1.28 ± 0.97 | 0.70± 0.59 | 1.1 ± 0.91 | 0.83 ± 0.78 | 0.28 | **0.027** | 0.899 | 0.09 |
| HE4 (pmol/L) ^e^ | 149.45±189.00 | 218.54±191.91 | 178.24±184.78 | 294.07±324.30 | 0.343 | 0.198 | 0.835 | 0.419 |

^a^ *BRCA1* mutation carriers versus *BRCA2* mutation carriers.

^b^ *BRCA1* mutation carriers versus non-*BRCA* carriers.

^c^ *BRCA2* mutation carriers versus non-*BRCA* carriers.

^d^ *BRCA* mutation carriers versus non-*BRCA* carriers.

^e^ Only calculated in 61 ovarian cancer patients.

Here, *P* values were calculated by the Wilcoxon rank sum test and adjusted by the FDR procedure; *P* value < 0.05 in bold.
