# Supplementary material for: Comprehensive analysis of serum tumor markers and BRCA1/2 germline mutations in Chinese ovarian cancer patients
Source: Mol Genet Genomic Med. 2019 Apr 10;7(6):e672. doi: 10.1002/mgg3.672 (PMC6565576; doi:10.1002/mgg3.672)
Supplement: Supplementary file 3 [file MGG3-7-e672-s003.docx]

Supporting Table 2. The clinicopathological characteristics of benign controls

| **Characteristics** | | **N (%)** |
| --- | --- | --- |
| **Age at diagnosis** | ≤ 40 | 65 (29.68%) |
|  | 41-50 | 89 (40.64%) |
|  | 51-60 | 38 (17.35%) |
|  | ≥ 61 | 27 (12.33%) |
|  | Mean | 45.37 |
| **Histological subtype** | cervix benign mass | 5 (2.28%) |
|  | cervix cyst | 2 (0.91%) |
|  | endometriosis | 21 (9.59%) |
|  | ovarian benign mass | 39 (17.81%) |
|  | ovarian cyst | 44 (20.09%) |
|  | ovary teratoma | 25 (11.42%) |
|  | oviduct benign mass | 4 (1.83%) |
|  | oviduct cyst | 7 (3.20%) |
|  | pelvic benign mass | 9 (4.11%) |
|  | uterine atypical hyperplasia | 2 (0.91%) |
|  | uterine fibroids | 61 (27.85%) |
