# Supplementary material for: Comprehensive analysis of serum tumor markers and BRCA1/2 germline mutations in Chinese ovarian cancer patients
Source: Mol Genet Genomic Med. 2019 Apr 10;7(6):e672. doi: 10.1002/mgg3.672 (PMC6565576; doi:10.1002/mgg3.672)
Supplement: Supplementary file 4 [file MGG3-7-e672-s004.docx]

Supporting Table 3. The statistics and associations between serum tumor markers and clinical characteristics in ovarian cancer patients

| **Characteristics** | | **N (%)** | **AFP (ng/mL)** | **CA125 (U/mL)** | **CA15-3 (U/mL)** | **CA19-9 (U/mL)** | **CA242 (U/mL)** | **CEA (ng/mL)** | **Ferritin (ng/mL)** | **HGH (ng/ml)** | **NSE (ng/mL)** | **β-HCG (ng/mL)** | **HE4 (pmol/L)^a^** | **HE4 N (%)^b^** |
| --- | --- | --- | --- | --- | --- | --- | --- | --- | --- | --- | --- | --- | --- | --- |
| **Age at diagnosis** | ≤ 40 | 27 (11.64%) | 44.08±99.49 | 246.82±278.58 | 20.61±41.34 | 54.32±101.4 | 19.26±42.27 | 3.84±7.97 | 108.92±167.8 | 1.29±2.01 | 7.5±13.99 | 0.76±0.61 | 98.44±161.47 | 6 (9.84%) |
|  | 41-50 | 85 (36.64%) | 3.24±4.67 | 251.67±261.59 | 16.59±20.02 | 51.42±133.57 | 14.09±30.16 | 2.86±7.8 | 92.58±87.85 | 0.38±0.82 | 5.27±4.84 | 0.72±0.7 | 197.1±237.91 | 23 (37.7%) |
|  | 51-60 | 71 (30.60%) | 2.65±3.92 | 328.08±320.25 | 22.92±22.9 | 32.31±90.89 | 11.94±26.14 | 3.72±14.86 | 134.24±109.51 | 0.29±0.58 | 7.37±6.91 | 1.03±0.9 | 322.85±324.49 | 20 (32.79%) |
|  | ≥ 61 | 49 (21.12%) | 6.11±21.33 | 354.85±310.55 | 19.67±25.91 | 55.53±111.31 | 15.48±33.8 | 4.19±11.13 | 195.4±115.43 | 0.28±0.55 | 7.26±5.86 | 1.09±0.91 | 413.97±378.2 | 12 (19.67%) |
|  | *P* value |  | 0.1194 | 0.2922 | 0.1932 | **0.0486** | 0.8936 | 0.7415 | **1.07E-06** | 0.1936 | 0.1194 | 0.0837 | 0.0837 |  |
| **Histological subtype** | Serous | 176 (75.86%) | 3.59±11.71 | 323.19±302.21 | 20.82±23.26 | 37.43±90.72 | 10.97±20.58 | 2.91±10.57 | 138.22±117.7 | 0.31±0.68 | 6.99±8.01 | 0.94±0.83 | 307.74±310.24 | 44 (72.13%) |
|  | Mucinous | 16 (6.90%) | 7.05±8.04 | 97.5±158.34 | 22.11±52.81 | 123.75±205.18 | 46.55±78.05 | 11.82±21.04 | 56.07±57.17 | 1.29±2.12 | 3.99±2.24 | 0.84±0.94 | 37.67±15.78 | 5 (8.2%) |
|  | Endometrioid | 7 (3.02%) | 3.95±5.12 | 375.82±290.67 | 25.35±28.82 | 175.48±294.25 | 32.15±48.62 | 1.74±2.01 | 88.92±74.9 | 1.23±1.83 | 5.16±4.15 | 0.99±0.76 | 467.9±368.48 | 3 (4.92%) |
|  | Clear cell | 7 (3.02%) | 3.5±2.64 | 150.14±244.68 | 5.71±3.88 | 25.77±35.95 | 6.68±4.83 | 1.5±1.31 | 112.48±101.9 | 0.34±0.49 | 3.99±1.62 | 0.5±0.66 | 125.92±123.99 | 3 (4.92%) |
|  | Others | 21 (9.05%) | 54.4±111.17 | 180.73±210.89 | 8.78±6.8 | 24.23±38.03 | 10.44±23.79 | 3.87±5.19 | 112.43±155.64 | 0.67±1.25 | 5.74±5.62 | 0.54±0.43 | 41.02±6.31 | 4 (6.56%) |
|  | unknown | 5 (2.16%) | 2.93±2.74 | 549.49±308.9 | 25.58±18.44 | 63.88±124.12 | 28.06±51.24 | 1.37±0.84 | 187.6±66.62 | 0.01±0 | 10.03±3.2 | 1.25±0.91 | 436.9±577.14 | 2 (3.28%) |
|  | *P* value |  | 0.3067 | **0.0117** | **0.0406** | 0.6888 | 0.8920 | 0.6762 | **0.0117** | 0.0575 | 0.2577 | 0.2440 | 0.1139 |  |
| **FIGO stage** | Ⅰ | 48 (20.69%) | 7.04±21.83 | 109.79±185.85 | 15.32±32.16 | 57.56±138.12 | 16.93±41.89 | 4.7±11.49 | 64.68±65.48 | 0.52±1 | 3.73±2.3 | 0.56±0.61 | 144.93±204.81 | 15 (24.59%) |
|  | II | 22 (9.48%) | 4.08±5.38 | 189.14±249.07 | 17.68±21.63 | 89.93±150.09 | 14.59±26.44 | 7.44±26.08 | 110.85±97.58 | 0.4±0.53 | 4.51±3.32 | 0.71±0.66 | 169.58±198.6 | 6 (9.84%) |
|  | III | 118 (50.86%) | 10.41±47.31 | 375.65±299.42 | 21.81±24.07 | 40.38±102.84 | 13.61±26.87 | 2.65±7.24 | 158.73±133.76 | 0.33±0.76 | 7.32±6.62 | 1.06±0.88 | 319.53±318.97 | 34 (55.74%) |
|  | IV | 25 (10.78%) | 12.05±41.6 | 291.26±272.1 | 19.02±25.64 | 45.75±107.15 | 19.51±43.94 | 3.27±5.02 | 111.1±92.25 | 0.7±1.33 | 9.97±14.92 | 0.87±0.75 | 778.5±111.02 | 2 (3.28%) |
|  | unknown | 19 (8.19%) | 1.64±1.42 | 397.01±328.1 | 19.98±18.46 | 11.35±11.47 | 6.19±4.54 | 1.69±1.3 | 145.92±97.83 | 0.62±1.82 | 7.7±7.68 | 0.96±0.83 | 233.99±407.39 | 4 (6.56%) |
|  | *P* value |  | 0.7571 | **8.95E-08** | 0.2274 | 0.1249 | 0.7249 | 0.9866 | **8.09E-05** | 0.2274 | **0.0024** | **0.0056** | 0.0864 |  |
| **Grade** | Low | 11 (4.74%) | 3.72±4.32 | 199.32±282.43 | 14.34±14.42 | 19.18±19.82 | 9.26±11.09 | 2.63±2.48 | 115.67±100.55 | 0.67±1.13 | 6.26±8.12 | 0.81±0.65 | 50.4±NA | 1 (1.64%) |
|  | Middle | 22 (9.48%) | 4.86±6.59 | 345.48±288.78 | 14.35±18.56 | 75.87±154.85 | 23.41±46.42 | 11.7±29.05 | 137.69±105.25 | 0.5±1.2 | 5.47±3.92 | 0.92±0.79 | 271.43±292.9 | 5 (8.2%) |
|  | High | 134 (57.76%) | 3.42±13.06 | 345.3±304.51 | 22.72±24.59 | 42.09±108.5 | 10.68±17.9 | 1.73±1.62 | 142.91±121.63 | 0.26±0.51 | 7.64±8.49 | 0.99±0.85 | 349.79±322.72 | 36 (59.02%) |
|  | unknown | 65 (28.02%) | 20.99±67.09 | 195.95±250.98 | 15.99±29.68 | 51.75±116.25 | 19.74±45.55 | 4.64±11.51 | 99.7±113.5 | 0.74±1.46 | 4.88±4.72 | 0.72±0.76 | 134.14±228.1 | 19 (31.15%) |
|  | *P* value |  | 0.1753 | **0.0287** | **0.0283** | 0.2946 | 0.8517 | 0.7404 | **0.0363** | 0.1915 | 0.0526 | 0.1765 | 0.1651 |  |
| **Lymph node metastasis** | Positive (+) | 98 (42.24%) | 8.58±38.54 | 266.39±303.23 | 17.43±24.54 | 55.84±132.62 | 14.05±30.77 | 3.74±12.66 | 123.08±120.44 | 0.47±1.06 | 5.92±5.87 | 0.82±0.81 | 305.11±328.41 | 20 (32.79%) |
|  | Negative (-) | 134 (57.76%) | 8.45±36.6 | 338.23±277.86 | 22.77±26.44 | 34.07±76.78 | 14.77±32.34 | 3.2±8.28 | 137.2±114.32 | 0.39±0.88 | 7.55±8.92 | 1±0.81 | 254.79±294.52 | 41 (67.21%) |
|  | *P* value |  | 0.1346 | 0.1077 | 0.1346 | 0.6275 | 0.2930 | 0.1647 | 0.1647 | 0.2056 | 0.1245 | 0.1077 | 0.5382 |  |
| **Marriage age** | unmarried | 11 (4.74%) | 56.72±120.32 | 246.41±305.03 | 9.58±8.03 | 22.59±13.3 | 6.21±2.73 | 1.85±1.8 | 78.74±142.69 | 1.85±2.46 | 3.62±1.73 | 0.56±0.49 | 59.09±37.34 | 4 (6.56%) |
|  | ≤20 | 76 (32.76%) | 4.89±17.31 | 362.55±306.72 | 22.2±22.01 | 60.91±143.65 | 13.91±24.8 | 3.57±14.18 | 143.94±113.17 | 0.24±0.42 | 7.39±6.39 | 1.06±0.89 | 253.42±318.81 | 17 (27.87%) |
|  | 21-23 | 68 (29.31%) | 11.07±44.21 | 262.82±289.29 | 20.41±32.7 | 49.04±101.15 | 15.36±36.21 | 4.89±12.45 | 118.47±113.45 | 0.57±1.19 | 5.8±6.04 | 0.87±0.85 | 353.13±286.83 | 15 (24.59%) |
|  | ≥ 24 | 61 (26.29%) | 2.81±3.49 | 300.15±290.51 | 19.97±24.95 | 23.09±59.13 | 10.9±25.16 | 2.22±4.41 | 142.18±127.64 | 0.32±0.64 | 7.26±10.15 | 0.83±0.73 | 300.3±330.68 | 22 (36.07%) |
|  | unkown | 16 (6.90%) | 4.12±6.6 | 126.1±174.27 | 9.62±8.9 | 82.85±175.91 | 32.4±59.21 | 4±10.03 | 80.52±79.11 | 0.29±0.58 | 5.4±4.1 | 0.69±0.69 | 33.43±10.36 | 3 (4.92%) |
|  | *P* value |  | 0.6217 | 0.1950 | 0.1950 | 0.3676 | 0.8708 | 0.8708 | 0.1455 | 0.3345 | 0.3676 | 0.3676 | 0.3266 |  |
| **Menstrual age** | ≤13 | 72 (31.03%) | 14.78±54.28 | 333.53±295.21 | 20.09±22.03 | 47.34±121.12 | 15.29±35.41 | 4.59±12.18 | 126.94±123.38 | 0.35±0.62 | 6.8±6.36 | 0.94±0.87 | 297.82±270.07 | 18 (29.51%) |
|  | 14-15 | 100 (43.10%) | 4.23±15.26 | 246.85±279.2 | 19.54±27.62 | 43.39±98.77 | 14.65±32.72 | 3.57±13.17 | 115.01±113.63 | 0.51±1.17 | 6.07±8.43 | 0.77±0.7 | 206.13±275.73 | 27 (44.26%) |
|  | ≥ 16 | 53 (22.84%) | 8.56±40.98 | 344.42±307.48 | 20.65±26.94 | 51.56±129.01 | 11.27±19.44 | 2.13±2.4 | 154.57±119.86 | 0.46±1.07 | 7.11±6.57 | 1.05±0.88 | 351.39±374.73 | 16 (26.23%) |
|  | unknown | 7 (3.02%) | 3.61±2.55 | 224±349.67 | 7.12±3.75 | 54.52±113.37 | 25.35±46.47 | 2.28±1.21 | 150.96±80.36 | 0.1±0.19 | 8.11±5.54 | 1.16±1.17 | - | 0(0%) |
|  | *P* value |  | 0.7582 | 0.4312 | 0.7582 | 0.9744 | 0.9262 | 0.7582 | 0.4312 | 0.9262 | 0.7462 | 0.7462 | 0.4733 |  |
| **Menopausal age** | unmenopausal | 70 (30.17%) | 16.05±60.71 | 294.58±282.59 | 21±31.77 | 40.36±99.84 | 15.02±35.16 | 4.02±9.66 | 87.88±118.9 | 0.84±1.55 | 5.61±5.19 | 0.82±0.73 | 170.07±244.67 | 16 (26.23%) |
|  | ≤45 | 41 (17.67%) | 8.56±30.97 | 287.37±290.8 | 19.78±20.42 | 49.18±132.65 | 13.33±25.28 | 1.82±1.45 | 125.3±105.9 | 0.34±0.5 | 7.1±11.52 | 0.79±0.72 | 231.85±270.59 | 9 (14.75%) |
|  | 46-50 | 67 (28.88%) | 4.11±18.12 | 298.33±295.02 | 16.2±15.96 | 30.98±70.88 | 8.1±11.63 | 1.88±2.61 | 147.48±119.44 | 0.22±0.5 | 6.54±6.48 | 0.87±0.83 | 294.34±299.35 | 23 (37.7%) |
|  | ≥ 51 | 43 (18.53%) | 3.88±5.28 | 315.77±323.92 | 23.68±31 | 67.29±138.77 | 22.09±45.79 | 7.2±21.9 | 160.4±108.49 | 0.28±0.49 | 7.45±6.85 | 1.1±0.93 | 382.37±381.59 | 13 (21.31%) |
|  | unkown | 11 (4.74%) | 3.85±3.47 | 239.51±313.26 | 14.89±17.34 | 107.41±204.74 | 23.35±37.77 | 1.98±1.07 | 181.4±117.72 | 0.07±0.16 | 8.39±5.92 | 1.23±1.1 | - | 0(0%) |
|  | *P* value |  | 0.5972 | 0.9725 | 0.8479 | 0.7349 | 0.7349 | 0.7802 | **9.46E-05** | 0.7349 | 0.7349 | 0.7802 | 0.7349 |  |
| **Fertility status** | ≤1 | 31 (13.36%) | 22.91±75.45 | 286.03±276.4 | 14.8±17.2 | 51.22±111.52 | 16.1±37.52 | 5.6±22.36 | 112.29±138.72 | 1.13±1.92 | 4.71±3.51 | 0.77±0.66 | 205.67±255.9 | 12 (19.67%) |
|  | 2月3日 | 120 (51.72%) | 5.01±18.44 | 293.94±305.56 | 20.22±27.85 | 38.42±87.97 | 13.05±29.79 | 3.69±9.75 | 114.96±98.25 | 0.33±0.66 | 6.26±5.97 | 0.87±0.82 | 276.74±292.79 | 31 (50.82%) |
|  | ≥ 4 | 75 (32.33%) | 8.68±38.46 | 315.78±291.62 | 21.17±24.88 | 61.03±147.98 | 16.28±32.39 | 2.52±4.81 | 159.43±134.82 | 0.34±0.78 | 8.04±9.97 | 1±0.87 | 320.65±363.83 | 17 (27.87%) |
|  | unkown | 6 (2.59%) | 2.93±2.17 | 117.81±125.31 | 12.56±9.53 | 8.52±5.55 | 5.8±5.84 | 1.88±1.33 | 107±77.79 | 0.12±0.2 | 4.48±2.35 | 0.76±0.68 | 50.4±NA | 1 (1.64%) |
|  | *P* value |  | 0.7138 | 0.7138 | 0.7138 | 0.4843 | 0.7138 | 0.7716 | 0.4843 | 0.5026 | 0.5026 | 0.7138 | 0.7716 |  |
| **Personal history of cancer** | Yes | 14 (6.03%) | 26.87±78.8 | 272.29±284.69 | 10.55±14.57 | 60.77±117.91 | 28.01±49.72 | 7.17±8.63 | 94.03±63.16 | 0.75±1.12 | 5.27±2.91 | 0.88±0.64 | 391.85±435.79 | 2 (3.28%) |
|  | No | 218 (93.97%) | 7.32±33.29 | 297.7±295.72 | 20.24±25.89 | 45.91±113.11 | 13.45±29.73 | 3.28±11.15 | 131.22±120.36 | 0.42±0.97 | 6.68±7.51 | 0.9±0.83 | 267.2±303.23 | 59 (96.72%) |
|  | *P* value |  | 0.1002 | 0.7977 | 0.2434 | 0.5859 | 0.4572 | **7.49E-04** | 0.6629 | 0.5614 | 0.7977 | 0.6629 | 0.5859 |  |

Note: The *P* values were calculated using the Wilcoxon rank sum test and corrected by the false discovery rate (FDR) procedure for multiple testing (Holm 1979). The corrected *P* value < 0.05 was in bold. ^a^ The statistics of HE4 level was calculated in 61 ovarian cancer patients; ^b^ The count statistics was only calculated in 61 ovarian cancer patients with HE4 level.
